# Supplementary material for: Evaluating Integrative Strategies for Incorporating Phenotypic Features in Spatial Transcriptomics
Source: ArXiv. 2025 Jul 29:arXiv:2507.22212v1. Preprint. [Version 1] (PMC12324568)
Supplement: Supplement 1 [file NIHPP2507.22212v1-supplement-1.pdf]

Supplementary Information

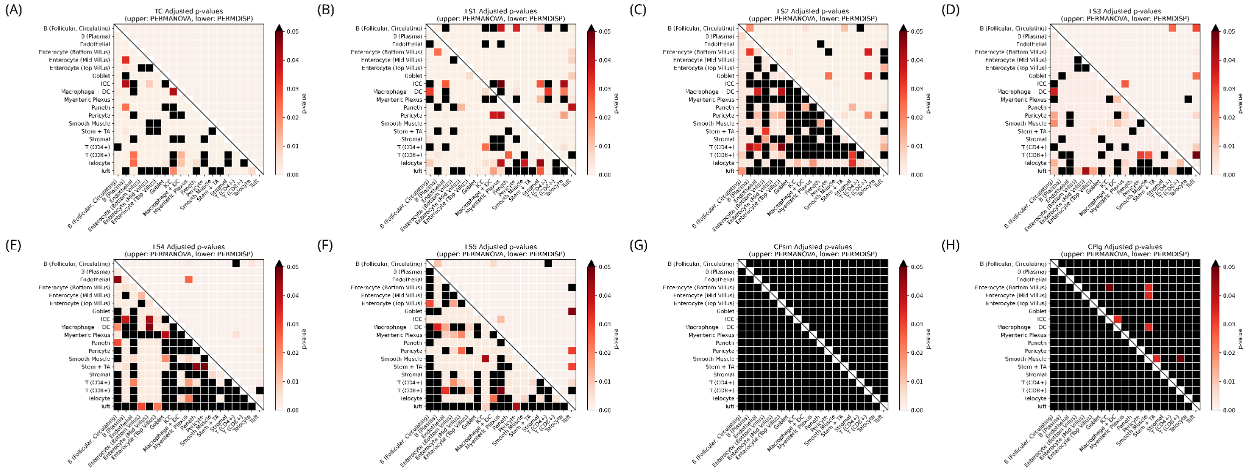

FIGURE S1 | Pairwise statistical comparisons between true labels in each feature space. (A–H) Half-matrix heatmaps showing pairwise permutational multivariate analysis of variance (PERMANOVA, upper triangle) and permutational analysis of multivariate dispersion (PERMDISP, lower triangle) results for each feature space: transcript count (TC), VAE latent spaces (LSs), and CellProfiler features from small (CPsm) and large (CPlg) crops. Multiple testing correction was applied using the Benjamini-Hochberg false discovery rate (FDR) method; p-values are color-mapped, with values above 0.05 shown in black.

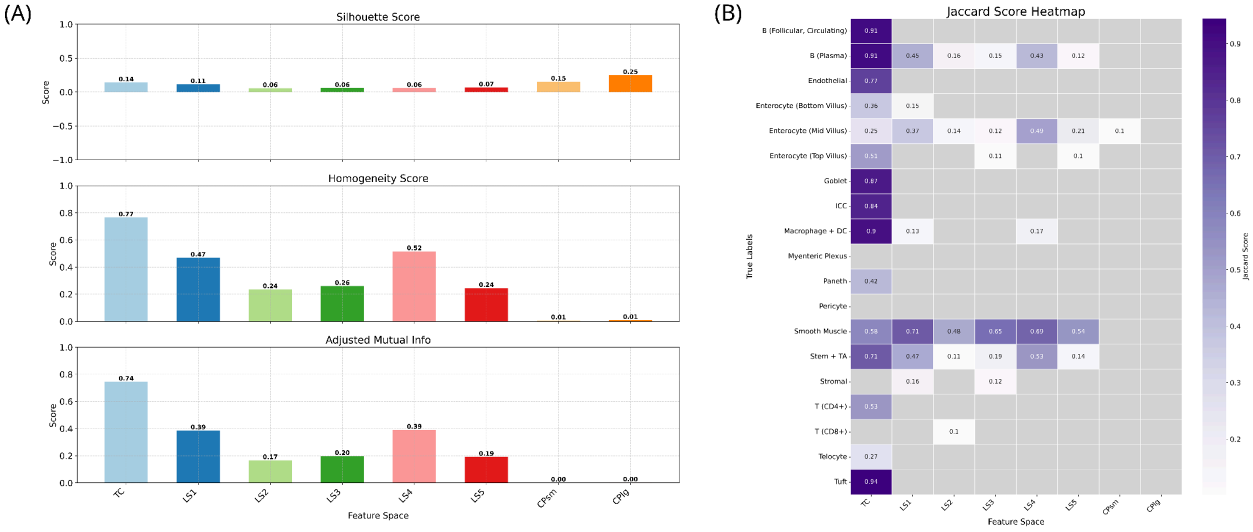

FIGURE S2 | Clustering metrics across the feature spaces. (A) Silhouette score (top), and homogeneity (middle) and adjusted mutual information scores (bottom) with respect to the ground truth partition. (B) Jaccard scores for clusters matched to true labels via the Hungarian algorithm across the feature spaces: transcript count (TC), VAE latent spaces (LSs), and CellProfiler features from small (CPsm) and large (CPlg) crops.

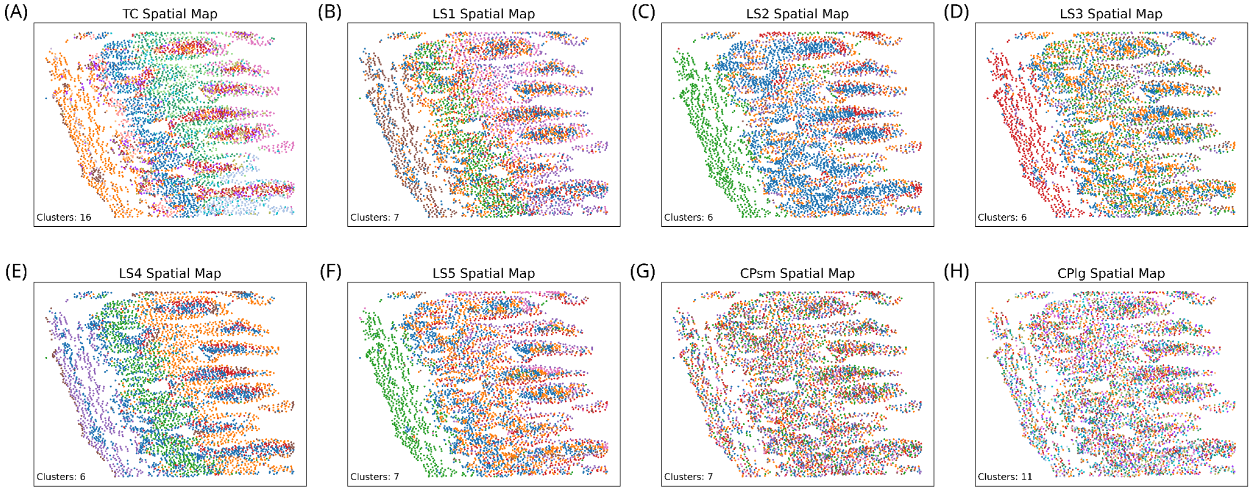

FIGURE S3 | Spatial organization of clustering results across feature spaces. (A–H) Spatial map of the tissue sample annotated by Leiden clustering results for individual feature spaces: transcript count (TC), VAE latent spaces (LSs), and CellProfiler features from small (CPsm) and large (CPlg) crops. The number of clusters is indicated in the bottom left of each panel.

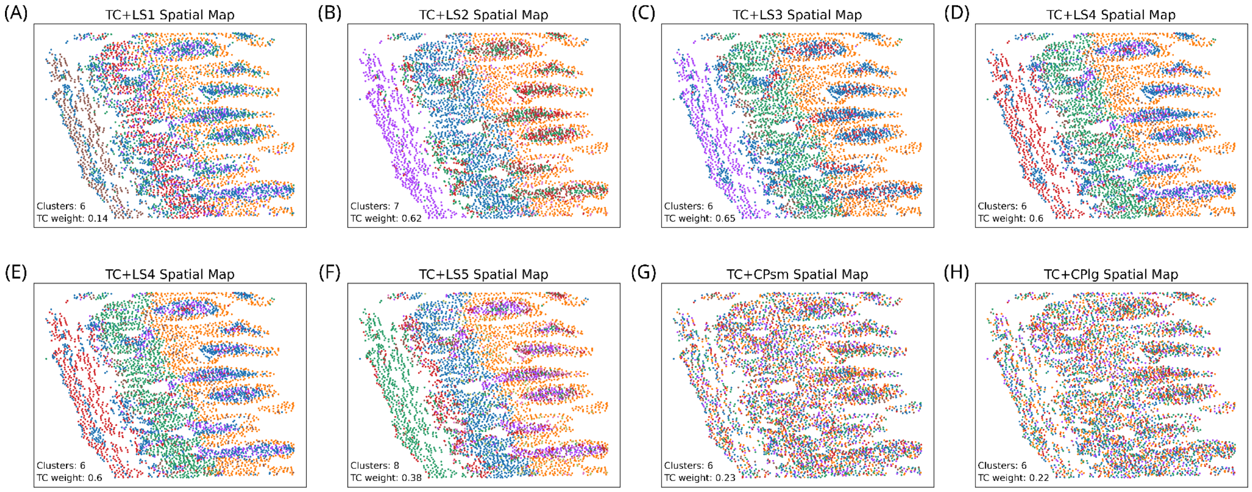

FIGURE S4 | Spatial organization of clustering results across multiplexed feature spaces. (A–H) Spatial map of the tissue sample annotated by Leiden clustering results for the transcript count (TC) feature space multiplexed with each VAE latent space (LS) or CellProfiler feature set from small (CPsm) and large (CPlg) crops. The number of clusters and the optimized TC modality weight (based on mean silhouette score) are indicated in each panel.

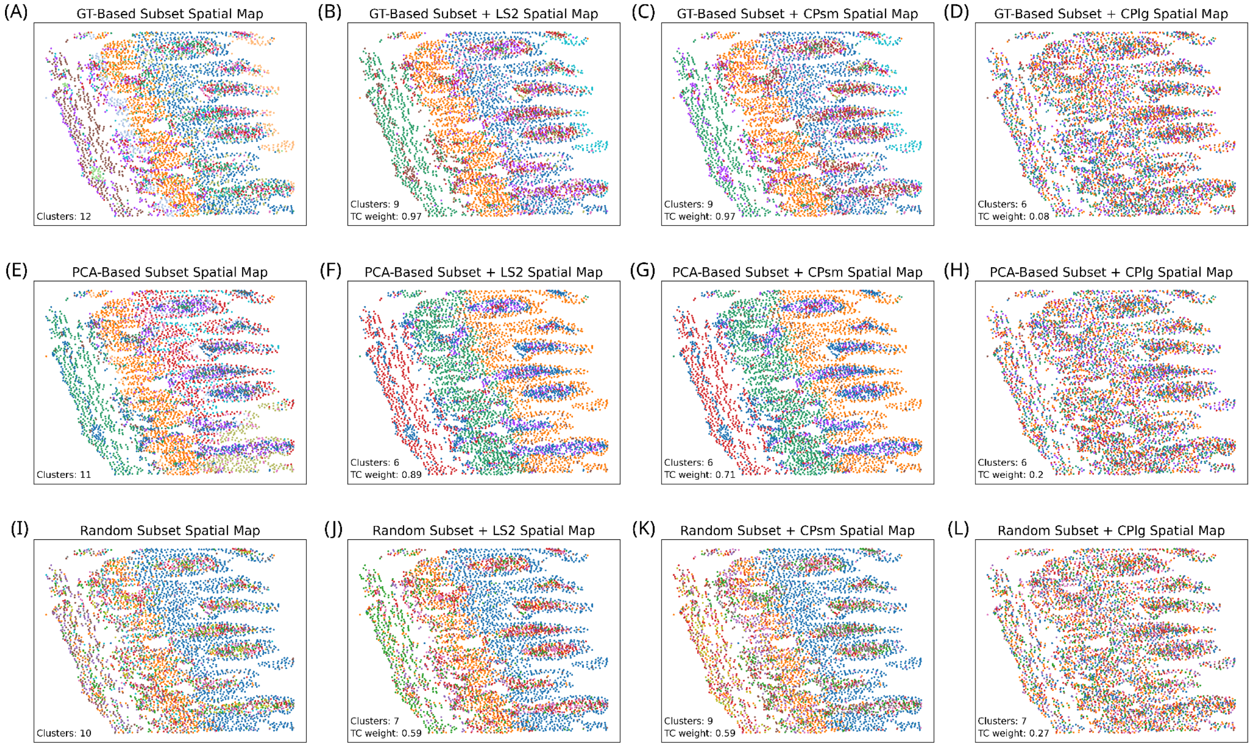

FIGURE S5 | Spatial organization of clustering results across multiplexed feature spaces using gene subsets. Spatial maps of the tissue sample annotated by multiplex Leiden clustering results. Rows correspond to different gene subset selection methods: (A–D) ground truth (GT)-based selection, (E–H) PCA-based selection, and (I–L) random selection. Each panel shows clustering based on the transcript count (TC) feature space multiplexed with the morphological latent space (LS2) or CellProfiler features from small (CPsm) or large (CPlg) crops. The number of clusters and the optimized TC modality weight (based on mean silhouette score) are indicated in each panel.
